# Neonatal hypoglycemia: lack of evidence for a safe management

**DOI:** 10.3389/fendo.2023.1179102

**Published:** 2023-06-08

**Authors:** Marcia Roeper, Henrike Hoermann, Sebastian Kummer, Thomas Meissner

**Affiliations:** Department of General Pediatrics, Neonatology and Pediatric Cardiology, Medical Faculty, University Hospital Düsseldorf, Heinrich-Heine-University, Düsseldorf, Germany

**Keywords:** neonatal hypoglycemia, brain damage, treatment threshold, at-risk neonates, treatment guidelines

## Abstract

Neonatal hypoglycemia affects up to 15% of all newborns. Despite the high prevalence there is no uniform definition of neonatal hypoglycemia, and existing guidelines differ significantly in terms of when and whom to screen for hypoglycemia, and where to set interventional thresholds and treatment goals. In this review, we discuss the difficulties to define hypoglycemia in neonates. Existing knowledge on different strategies to approach this problem will be reviewed with a focus on long-term neurodevelopmental outcome studies and results of interventional trials. Furthermore, we compare existing guidelines on the screening and management of neonatal hypoglycemia. We summarize that evidence-based knowledge about whom to screen, how to screen, and how to manage neonatal hypoglycemia is limited – particularly regarding operational thresholds (single values at which to intervene) and treatment goals (what blood glucose to aim for) to reliably prevent neurodevelopmental sequelae. These research gaps need to be addressed in future studies, systematically comparing different management strategies to progressively optimize the balance between prevention of neurodevelopmental sequelae and the burden of diagnostic or therapeutic procedures. Unfortunately, such studies are exceptionally challenging because they require large numbers of participants to be followed for years, as mild but relevant neurological consequences may not become apparent until mid-childhood or even later. Until there is clear, reproducible evidence on what blood glucose levels may be tolerated without negative impact, the operational threshold needs to include some safety margin to prevent potential long-term neurocognitive impairment from outweighing the short-term burden of hypoglycemia prevention during neonatal period.

## Introduction: neonatal glycemia – what is normal?

For a variety of reasons, the definition of “hypoglycemia” in newborns is more difficult than in adults. In adults, low blood glucose is considered “hypoglycemia” if blood glucose is sufficiently low to cause adrenergic or neuroglycopenic symptoms. Further evaluation or treatment of low glucose values is only recommended if the so-called Whipple’s triad is fulfilled (1, 2): 1) the development of autonomic or neuroglycopenic symptoms, 2) a low blood glucose level, and 3), prompt relief of symptoms when blood glucose has risen. As numeric definition, e.g. for clinical studies, a “low glucose” concentration <3.0 mmol/l (<54 mg/dl) was considered a clinically significant hypoglycemia as this is in a range where autonomic and/or neuroglycopenic symptoms are known to occur, however with high intra- and interindividual variability (3).

In newborns, a definition including clinical signs as hallmark cannot be applied, as they are neither specific nor sensitive at this stage of life, even at very low blood glucose levels (4). In addition, there is a physiological, transient drop of blood glucose levels during the first hours and days of life, complicating numeric definitions. Furthermore, the management of blood glucose in the neonatal period does not only need to consider what is “normal”, but also what is safe – particularly in terms of adverse neurodevelopmental outcomes, which is still a controversial issue (5–7).

### Definition of neonatal hypoglycemia - statistical approach

Quantitative laboratory parameters are usually assessed using population-based reference data, assuming a condition can be considered pathologic if its occurrence is statistically rare, e.g., outside of two standard deviations (~ <2.5^th^ or >97.5^th^ percentile), or <5^th^/>95^th^ percentile. From an evolutionary perspective, harmful conditions can be expected to occur rather rarely because an adaptation to the environment has taken place. With respect to neonatal hypoglycemia, an epidemiological approach means that we assume that the postnatal metabolism with respect to neonatal hypoglycemia evolved such that under normal circumstances, only a small number of newborns should exhibit relevant hypoglycemic damage. It may be doubted that this perspective is true for neonatal hypoglycemia, as providing all newborns with the best possible care and thus enabling them to develop as optimally as possible may require different threshold values than a plain statistical definition can offer.

The situation is further complicated by the fact that not only the individual low blood glucose value is important, but also the duration of an episode with low values is relevant for an insufficient energy supply, which affects the newborn and may lead to brain damage. Additionally, repeated episodes of low blood glucose levels, leading to a depletion of the low energy stores in the brain are also not captured by a statistical approach of defining a single glucose value as clinically relevant hypoglycemia. Thus, the pathologic relevance or potential for harm is therefore likely to be a continuum and not dichotomous, with mild hypoglycemia having limited or no impact and the risk of adverse consequences gradually increasing with the severity and duration of hypoglycemia (8, 9). Finally, it remains unclear whether a normal range for healthy-term newborns can be extrapolated to newborns at-risk.

A statistical definition must take into account that postnatal blood glucose levels are crucially dependent on postnatal food intake (10, 11). Therefore, reference levels also depend on breastfeeding habits, and the availability of breast/formula milk. Ideally, only studies that followed reasonable postnatal breastfeeding or formula feeding practices in the first hours of life should be considered as reference data, as these are more reflective of how blood glucose *should* be with adequate postnatal feeding.

For healthy term newborns, there are different data on the course of glucose concentrations during the first days of life. Srinivasan et al. published the blood glucose concentrations of 344 healthy full term appropriate for gestational age (AGA) infants. The lower estimate for the 95% confidence interval (CI) was 1.4 mmol/l (26 mg/dl) at one hour of life, increasing to 2.3 mmol/l (42 mg/dl) at two hours. Already at 3 hours of age, glucose concentration was significantly higher than at 1 hour of age, even without initiating of feeding. The authors concluded that glucose levels <1.9 mmol/l (<35 mg/dl) should be of concern in the first 3 hours of life, <2.2 mmol/l (<40 mg/dl) from 3-24 hours of life, and <2.5 mmol/l (<45 mg/dl) subsequently (12).

In contrast, a study from India found no significant variation in the blood glucose concentration between 3 and 72 hours of life in 200 healthy term neonates. Low glucose concentration was defined as <2.2 mmol/l (<40 mg/dl) for the first 24 hours of life and <2.5 mmol/l (<45 mg/dl) at 24-72 hours of life. Of note, the average birth weight of the cohort was only 2650 g, which is significantly lower than the average birth weight of infants of European decent, and all infants were exclusively and frequently breastfed at intervals of 1.5 to 2 hour from 6 hours of life. No differences in glucose concentration were observed with respect to time since last breastfeeding with this regimen. In 27 of 800 measurements, glucose concentrations were below 1.6 mmol/l (29 mg/dl) but higher than 1.3 mmol/l (23 mg/dl). Only one measurement revealed a level <1.3 mmol/l (<23mg/dl), and all infants were asymptomatic and attained “euglycemic levels” after feeding. Of note, low glucose levels <1.6 mmol/l (<29 mg/dl) were noted primarily around the 3rd hour of life but also after 72 hours of life, and a low glucose at 3 hours increased the risk for low glucose at 72 hours (risk ratio (RR) 6.55 [95% CI 3.93 – 10.92], p < 0.00001) (13). Alkalay et al. pooled the data of six selected studies and defined thresholds of low blood glucose corresponding to the 5^th^ percentile for different time intervals based on 723 term newborns. The lower estimated <5^th^ percentiles were <1.6 mmol/l (<28 mg/dl) (1-2h); <2.2 mmol/l (<40 mg/dl) (3-23h); <2.3 mmol/l (<41 mg/dl) (24-47h); <2.7 mmol/l (<48 mg/dl) (>48h), and were considered pragmatic operational thresholds, values at which a reaction is recommended (14). The concept of operational thresholds is discussed below.

In a recent study, Harris et al. examined longitudinal neonatal glucose concentrations in 67 healthy, term-born, AGA singletons in New Zealand by continuous glucose monitoring and repeated heel-prick blood glucose measurements from birth to 120 hours. Most of the infants were exclusively breast-fed. They noted an increase in mean glucose concentration during the first 18 hours, followed by a second phase in which glucose levels remained stable in the same range up to 48 hours, followed by an increase to a new plateau by the fourth day. Blood glucose concentrations of 2.6 mmol/l (47 mg/dl), often used as a threshold, corresponded approximately to the 10^th^ percentile in the first 48 hours. Of the neonates for whom complete interstitial glucose data were available, 73% had at least one episode below this threshold, most within the first 12 hours. Using an interstitial glucose concentration threshold of <2.0 mmol/l (<36 mg/dl), approximately 25% of neonates were found to be below this level at least for one episode. Most of these lower interstitial or blood glucose concentrations occurred within the first 12 hours. There were no episodes of blood glucose concentrations of <1.5 mmol/L (<27 mg/dl) and only two episodes below this cut-off documented by continuous interstitial glucose measurements (15).

From this, despite all limitations as influence of ethnic background, feeding habits, glucose measurement intervals or definition of a “normal” percentile, it can be deduced that single measurements or short episodes of glucose concentrations less than 2.0 mmol/l (36 mg/dl) are “normal” using the statistical approach, while glucose concentrations of <1.3 – 1.5 mmol/l (<23 – 27 mg/dl) should be interpreted as unusual for healthy term newborns”.

### Approach to define hypoglycemia based on short-term physiological consequences of hypoglycemia (endocrine/metabolic responses, signs/symptoms)

This approach assumes that a low blood glucose should be primarily considered unphysiological if it leads to certain endocrine, metabolic, or neurological consequences/symptoms. Systematic data on counterregulatory mechanisms in neonates are scarce. Stanley et al. found low ketones despite elevated concentrations of fatty acid precursors, indicating limited ketone synthesis capacity in 44 healthy term AGA infants at the end of an eight-hour postnatal fast. Glucose precursors were two to three times higher than those found after the neonatal period, indicating immature gluconeogenesis capacity (9). Similar results were obtained by Harris et al. who found low ketone body concentrations but high lactate values in newborns at low glucose levels (16). However, an alternative hypothesis is that in the fasting situation or low glucose state, the neonatal brain consumes both glucose from gluconeogenesis and ketones to such a high degree that distinct different metabolite concentrations are found than in older fasting children, so capacity of ketogenesis and gluconeogenesis might be underestimated by levels of circulating ketones/glucose. During insufficient glucose supply of the brain, the glycogen of the astrocytes is presumably used to supply the neurons, being first converted into lactate, then transported to the neurons to maintain the neuronal function (17). In addition, blood lactate and ketones are taken up by neurons through monocarboxylate transporters (17).

In summary, the energy supply of neurons can be substituted at least in part by other metabolites than glucose. However, the approach of evaluating these metabolic or endocrinological parameters is also not useful in determining at which blood glucose levels hypoglycemia can be defined or brain damage is imminent.

Outcomes of symptomatic hypoglycemia in newborns have been shown to be worse than asymptomatic hypoglycemia (18, 19). However, we have recently shown that clinical signs are unspecific and not sensitive enough to reliably detect neonatal hypoglycemia based on clinical observation. We found a large interobserver differences, and even in the presence of profound hypoglycemia, the sensitivity to detect hypoglycemia based on clinical signs was rather low (4). Furthermore, severe hypoglycemia may present as apathy or coma, which are difficult to distinguish from deep sleep.

### Approach to define hypoglycemia based on neurological function or long-term neurological outcome

This approach attempts to find a threshold that allows undisturbed neurological function and development. Values associated with impaired neurological function or neurodevelopment would then be defined as “hypoglycemia”.

In 1988, Koh et al. measured sensory evoked potentials as a noninvasive indicator of brain function in relation to blood glucose concentration in 17 children, 5 of whom were newborns. The lowest blood glucose concentration associated with normal neural function in the newborns ranged from 1.9 to 4.2 mmol/l (34 - 76 mg/dl). The blood glucose concentration immediately before the first abnormal evoked potential ranged from 0.7 to 2.5 mmol/l (13 - 45 mg/dl) in these five newborns. The authors suggested that “the blood glucose concentration should be maintained above 2.6 mmol/l (47 mg/dl) to ensure normal neural function in children irrespective of the presence or absence of abnormal clinical signs” (20). As such low proband numbers warrant cautious interpretation, it does not seem appropriate that this study is repeatedly used to justify a hypoglycemia threshold of 2.6 mmol/l (47 mg/dl).

Neuroimaging studies have identified structural cerebral injury associated with severe, recurrent or symptomatic neonatal hypoglycemia, including white matter lesions preferentially in the parieto-occipital lobes, cortical atrophy, changes in the deep grey matter structures of the basal ganglia and thalamus, periventricular lesions, parenchymal hemorrhage, and ischemic strokes (21–30). Recent studies have associated also mild neonatal hypoglycemia <2.6 mmol/l (<47 mg/dl) in otherwise healthy children with a reduced size of deep grey matter brain regions and thinner occipital lobe cortex at the age of 9-10 years, but no differences regarding white matter microstructure were found. Therefore, the authors concluded that deep grey matter regions may be especially vulnerable to the long-term effects of mild neonatal hypoglycemia (31).

Clinical manifestations of hypoglycemic brain injury include cerebral palsy, epilepsy, neurodevelopmental delay and intellectual disability, microcephaly, visual impairment, and hearing deficits (32). In hypoglycemic disorders such as congenital hyperinsulinism the reported incidence of brain injury due to severe and recurrent hypoglycemia is still as high as 50% (32–36).

While there is no doubt that severe hypoglycemia of any etiology can lead to brain injury, the effects of mild and transitory neonatal hypoglycemia remain unclear.

Adverse clinical outcome after transitory neonatal hypoglycemia was first described 1988 in a larger study of 661 preterm infants with birth weights <1850 g, reporting that blood glucose concentrations <2.6 mmol/l (<47 mg/dl) for five or more days, even if asymptomatic, were associated with serious neurodevelopmental impairment at a corrected age of 18 months (37). Even though this study had limitations, such as including only preterm infants with e.g., immature counterregulatory response to hypoglycemia, the aim to develop a definition of neonatal hypoglycemia became more urgent. As a consequence of the study, a threshold of 2.6 mmol/l (47 mg/dl) has since been considered a target for the treatment of neonatal hypoglycemia by many neonatologists. However, since 1988, several other studies have examined the neurodevelopmental outcome after neonatal hypoglycemia with contradictory results. In 2019, Shah et al. evaluated nine cohort studies involving 4,041 infants with a gestational age >32 weeks in a systematic review. They concluded that there was low-quality evidence that neonatal hypoglycemia <2.6 mmol/l (<47 mg/dl) was associated with a two- to threefold increased risk of visual-motor impairment and executive dysfunction in early childhood (2–5 years), and a twofold increased risk of literacy and numeracy problems in later childhood (6–11 years). Evidence for an increased risk of general cognitive impairment was rated as very low-quality, and no data was found on the outcome in adolescence with prior neonatal hypoglycemia (38).

One study included in the review was the ‘Children With Hypoglycemia and Their Later Development (CHYLD) Study’, a longitudinal prospective cohort study investigating neurodevelopmental outcomes in moderate to late preterm and term infants born at risk of hypoglycemia and treated to maintain blood glucose concentrations above 2.6 mmol/l (47 mg/dl). While this study found no association between neonatal hypoglycemia <2.6 mmol/l (<47 mg/dl) and an adverse neurological outcome at two years of age (39), neonatal hypoglycemia was correlated with an increased risk of poor executive function (RR 2.32 [95% confidence interval (CI) 1.17 - 4.59] and poor visual motor function (RR 3.67 [95% CI 1.15 - 11.69] at the age of 4.5 years (40). At 9-10 years, there was no significant difference between children with and without neonatal hypoglycemia regarding the incidence of lower educational achievement (47% vs. 48%; adjusted risk difference −2% [95% CI, −11% to 8%]; adjusted RR 0.95 [95% CI, 0.78 to 1.15]) and other secondary endpoints. However, the reported incidence of low performance overall, including educational achievement, fine-motor and visual-motor functions, emotional behavior regulation and executive function, were concerningly high in both groups and over twice as high as rates expected by the investigators. They concluded that the underlying risk factors for neonatal hypoglycemia and the socioeconomic status rather than hypoglycemia itself may play a greater role in the neurodevelopmental outcome than previously assumed (41). Two other recent studies also did not find any relevant adverse outcome associated with neonatal hypoglycemia if treated to maintain blood glucose concentrations above a certain threshold: In a Danish study, neonatal hypoglycemia <1.7 mmol/l (<30 mg/dl), treated to maintain blood glucose concentrations above 2.5 mmol/l (45 mg/dl), was solely associated with lower fine-motor function in boys (β = - 16.4, p = 0.048) compared to healthy siblings at age 6-9 years. The authors did not find any association between neonatal hypoglycemia and cognitive function, general motor function or behavior. However, the full-scale IQ was 3.2 points lower in the case group compared to the normative population and a major limitation was the low number of sibling controls (n=32) (42).

The HypoEXIT (Hypoglycemia–Expectant Monitoring versus Intensive Treatment) trial was a multicenter, randomized, controlled trial, analyzing noninferiority of a lower treatment threshold strategy (<2.0 mmol/l; <36 mg/dl) to the traditional threshold strategy (<2.6 mmol/l; <47 mg/dl). The operational threshold was defined based on two treatment arms: The intensive treatment arm aimed to rapidly achieve blood glucose concentrations >2.6 mmol/l (>47 mg/dl) by increasing the carbohydrate intake by enteral or intravenous glucose supply. In the expectant glucose monitoring arm, oral nutrition was given aiming at glucose concentrations always >2.0 mmol/l (>36 mg/dl). A glucose concentration below this resulted in intensive treatment. If the first low glucose concentration was <2.0 mmol/l (<36 mg/dl), the infant was excluded from the study and received intensive treatment.

Developmental testing using the Bayley Scales of Infant and Toddler Development in 582 children at the age of 18 months did not cross the prespecified noninferiority limit of −7.5 Bayley-test points for the lower threshold group. However, recurrent or severe hypoglycemia was associated with a worse neurological outcome at follow-up. A limitation of the study is certainly that neonates with initial severe hypoglycemia <2.0 mmol/l (<36 mg/dl) were excluded, the very patients most vulnerable to suffer consequences of hypoglycemia. Furthermore, despite the different intervention thresholds, the mean glucose values during the first two days were quite similar in the two comparative groups (3.2 vs. 3.4 mmol/l; 57 mg/dl vs. 61 mg/dl; mean difference -4.4 [-5.6 to -3.1] (43). Another important limitation is that the patients were quite young at neurodevelopmental follow-up and the long-term data on neurological outcome are not yet available.

Despite these data indicating non-inferior outcome of neonates who suffered neonatal hypoglycemia between 2.0 - 2.6 mmol/l (36 - 47 mg/dl), there are contradicting data. Kaiser et al. matched perinatal data from 1395 children, who had received a universal glucose screening after birth, to their Arkansas Department of Education’s fourth-grade achievement test scores. Three cut-offs for hypoglycemia were used (<1.9, <2.2, and <2.5 mmol/l; <35, <40, and <45 mg/dl) and data were controlled for covariables. Even one single hypoglycemic episode <2.5 mmol/l (<45 mg/dl) was associated with decreased school proficiency in literacy (odds ratio (OR) 0.62 [95% CI 0.45 - 0.85] and episodes < 2.2 mmol/l (<40 mg/dl) were associated with decreased proficiency in mathematics (OR 0.51 [95% CI 0.34 – 0.78] at the age of 10 years (44).

In a retrospective population-based study published in 2018, register data of all 101,060 healthy singletons born in two Swedish counties over a period of 4.5 years were screened using International Classification of Diseases 10 (ICD-10) codes, linking a diagnosis of transitory hypoglycemia <2.2 mmol/l (<40 mg/dl) to prespecified neurologic or developmental diagnoses. The OR of any adverse neurological or neurodevelopmental outcome was 1.48 [95% CI 1.17 - 1.88] in hypoglycemic compared to euglycemic newborns. Furthermore, those with a history of neonatal hypoglycemia had an almost doubled risk of motor delay (OR 1.91 [95% Cl 1.06 - 3.44] and a tripled risk of cognitive developmental delay (OR 3.17 [95% 1.35 - 7.43] (45). In summary, there is also insufficient evidence and too much controversy to define neonatal hypoglycemia based on neurodevelopmental outcome alone. The most valid data available at this time are from the CHYLD study (39–41) as well as the HypoEXIT trial (43), but further studies are needed.

### How to integrate different approaches to define hypoglycemia - the operational threshold

Thirty-five years after the publication by Lucas et al. (37), there are still no data available that define how low a glucose concentration must be, respectively how long it must persist at which level to cause brain injury in neonates. Thus, the concept of an “operational threshold” was proposed by Cornblath et al. (46). Operational thresholds should be single blood glucose concentrations at which therapeutic interventions are recommended, to prevent at least those blood glucose concentrations that have a clinically relevant *probability* of causing harm. As such, it will always imply some degree of over- and undertreatment, which needs to be balanced, and does not necessarily follow strict statistical evidence. An operational threshold may be different from a “treatment target”, that might be higher and both values may imply some safety margin to reliably prevent blood glucose concentration at which “organ damage is known to occur” (47). Furthermore, the interventional threshold does not need be based on blood glucose levels alone but may also be a composite of blood glucose levels, clinical signs, risk factors for hypoglycemia, presence of symptoms, fasting or satiety state, presumed neurological consequences etc., all of which contribute to therapeutic/interventional decisions. The following section outlines how the approach and handling of different operational thresholds varies in guidelines. These operational thresholds need to be further evaluated in future studies to provide evidence for the recommended interventions so that they are not based solely on expert opinion but rather on research data.

## Practical application of the operational threshold: existing guidelines for detection and management of neonatal hypoglycemia

In 1988, Koh et al. found that there was no uniform definition of neonatal hypoglycemia among established pediatric textbooks and or neonatologists in the UK, ranging from a glucose concentration of <1 to <4 mmol/l (<18 to <72 mg/dl). He concluded that this certainly lead to confusion among junior medical and nursing staff and inconsistency in the management of neonatal glycemia (48). In 2019, there continued to be large differences in knowledge about prevention, screening and management of neonatal hypoglycemia among midwives and nurses in Germany (49). Until today, there is still no uniform international guideline for detection and management of neonatal hypoglycemia, however, national guidelines exist in several countries. While some even have multiple guidelines, as for example Australia (50–52), other countries such as Germany do not have a general guideline for neonatal hypoglycemia, but only one for the management of children of diabetic mothers (53). Most guidelines admit that they are based on poor evidence and that there is no consensus on the definition of hypoglycemia and operational thresholds (5, 50, 51, 54–60). UNICEF (United Kingdom) has published a document called “Guidance on the development of policies and guidelines for the prevention and management of hypoglycaemia of the newborn” (61). showing that the topic is highly relevant and some of the existing guidelines refer to this publication as well (54, 56, 60, 62). An exemplary list of published guidelines showing the varying recommendations on who should be screened, when the first blood glucose should be measured, how hypoglycemia is defined, etc. is shown in **Table 1**. The comparability of glucose values is complicated by the fact that some guidelines refer to blood glucose and others to plasma glucose. For unification, we refer to blood glucose in this manuscript.

**Table 1:** Comparison of international guidelines for screening and management of neonatal hypoglycemia.

	Screening recommended		Time of first BG	Hypoglycemia threshold <48/72 h of life	Management based on asymptomatic/symptomatic?	Start of i.v. glucose	Further measures included	End of BG screening
	Neonatal risk factors	Maternal risk factors						
Hypoglycemia -newborn, Maternity and Neonatal Clinical Guideline (06/2022, Queensland, Australia) (51)	- Preterm infants (GA <37 weeks) - Postmature infants (GA >42 weeks) - SGA (<10^th^ centile) or BW <2500 g - LGA (>90^th^ centile) or macrosomia - Hypothermia: <36.5 °C or labile - Inadequate feeding - Resuscitation at birth - Polycythemia - Meconium aspiration syndrome - Suspected syndromes - Symptomatic	- Infants of mothers with diabetes - Maternal beta-blockers, dexamethasone, oral hypoglycemics - Family history of metabolic and/or endocrine disorders	Before the 2^nd^ feed (not longer than 3 h of age)	<48 h: <47 mg/dl (<2.6 mmol/l)	Yes	<27 mg/dl (<1.5 mmol/l) or symptomatic	Buccal glucose gel 40% Glucagon i.v. (also continuously)	BG ≥47 mg/dl (≥2.6 mmol/l) for 24 h in 1^st^ 48 h or ≥60 mg/dl (≥3.3) after 48 h
Child Women & Family Services Special Care Baby Unit, Waitemata District Health Board (07/2021, New Zealand) (62)	- Preterm infants (GA <37 weeks) - BW <2.5 kg or SGA (BW <10^th^ centile) - BW >4.5 kg - Hypothermia - Apgar score <7 at 5 minutes - Neonates with hemolytic disease - Neonatal syndromes (e.g., BWS) - All infants with clinical signs - In SCBU, at least daily BG testing on all infants on i.v. fluids	- Diabetes in pregnancy - Maternal drug treatment (e.g., Propranolol, Prozac (Fluoxetine), illicit drug abuse)	At 1-2 h of age	<47 mg/dl (<2.6 mmol/l) (time range not defined)	No	<31 mg/dl (<1.7 mmol/l) 31-45 mg/dl (1.7-2.5 mmol/l): Feeding or i.v. dextrose 10%	Buccal dextrose gel Glucagon	If feeding well, monitor at least for 12 h. Any recorded hypoglycemia, monitor glucose for at least 12 h after last low level
BM Clinical Protocol #1: Guidelines for Glucose Monitoring and Treatment of Hypoglycemia in Term and Late Preterm Neonates (04/2021, U.S.) (56)	- Preterm infants (GA <35 weeks or late preterm infants with clinical signs or extremely poor feeding) - IUGR or marked wasting - BW <2.500 g or SGA <10^th^ centile for weight - Clinically evident wasting of fat + muscle bulk - LGA (>90^th^ centile & macrosomic appearance) - Discordant twin; weight 10% <larger twin - Perinatal stress; severe acidosis or hypoxia–ischemia - Cold stress - Polycythemia - Erythroblastosis fetalis - BWS - Microphallus or midline defect (indicating an underlying endocrine condition) - Suspected infection - Respiratory distress - Known or suspected inborn errors of metabolism or endocrine disorders - Any infant admitted to the NICU - Clinical signs of hypoglycemia	- Maternal diabetes or abnormal result of glucose tolerance test, especially if poorly controlled - Pre-eclampsia and pregnancy induced, or essential hypertension - Previous macrocosmic infants (as a proxy for undiagnosed diabetes in pregnancy) - Substance abuse - Treatment with beta-agonist tocolytic - Treatment with oral hypoglycemic agents - Late antepartum or intrapartum administration of i.v. glucose	Infants with suspected significant hyperinsulinemia (e.g., poorly controlled maternal diabetes or known genetic hyperinsulinemia: within 60 min after birth. Other risk groups: Before the 2^nd^ feed, or 2-4 h after birth	<45 mg/dl (<2.5 mmol/l) (time range not defined)	Yes	If BG is repeatedly <45 mg/dl (<2.5 mmol/l) despite feedings If the neonate is unable to suck or feedings are not tolerated, avoid forced feedings and begin IV therapy Infants with abnormal clinical signs, or infants with BG levels <20-25mg/d (<1.1-1.4 mmol/l)	Buccal dextrose gel	Monitoring should continue until acceptable pre-prandial levels are consistently obtained (until the infant has had at least 3 satisfactory BG). A reasonable (although arbitrary) goal is to maintain BG concentrations ≥45 mg/dl (≥2.5 mmol/l). If energy intake falls, glucose monitoring should be recommenced. Late preterm and SGA infants and babies who have clinical features of intrauterine growth restriction should be monitored (with decreasing frequency) for 24 h
Guideline Hypoglycemia (02/2021, Western Australia) (52)	- Preterm infants (GA <37 weeks) - SGA (<10^th^ centile) - LGA (>97^th^ centile or >4.5 kg) - Beta-blockers in the 3rd trimester	- Maternal diabetes - Antenatal corticosteroids >34 weeks gestation	Before the 2^nd^ feed (3-4 h of age)	<47 mg/dl (<2.6 mmol/l) (time range not defined)	Yes	<27 mg/dl (1.5 mmol/l) or if BG remains between 27-45 mg/dl (1.5-2.5 mmol/l) despite increased feeds	Glucagon i.m.	If 2 consecutive BG are ≥47 mg/dl (≥2.6mmol/l)
Prevention and treatment of hypoglycemia in neonates with a gestational age from 35 0/7 weeks in maternity wards (09/2020, Switzerland) (57)	- Preterm infants (GA<37 0/7 weeks) - BW <2500 g or <3^rd^ centile - BW >4500 g or >97^th^ centile - Hypothermia <36.5°C - Sick newborn infants (e.g., asphyxia, sepsis, respiratory distress, hemolysis) - Symptomatic	- Maternal diabetes (including both, women treated only with dietary intervention and those receiving insulin)	Before the 2^nd^ feed (age 3-4 h)	<47 mg/dl (<2.6 mmol/l) (time range not defined)	No	Not described in detail ➔ only contact neonatal unit	Dextrose gel 40%	If 3 consecutive BG are normal, further blood tests may be discontinued
South Australian Perinatal Practice Guideline, Neonatal Hypoglycemia (09/2020, South Australia) (50)	- Preterm infants (GA ≥32 weeks) - BW <10^th^ centile - BW >97^th^ centile - Transient tachypnea of the newborn - Hypothermia <36.0°C - Suspected asphyxia requiring IPPV or APGAR <6 at 5 min or pH <7.1	- Maternal diabetes - Maternal beta-blockers or valproate	At 1 h of age	1-4 h: ≤36 mg/dl (≤2.0 mmol/l) 5-48 h: <47 mg/dl (<2.6 mmol/l)	Yes, but only for severe symptoms (seizures, critically unwell etc.)	<27 mg/dl (<1.5 mmol/l) <47 mg/dl (<2.6 mmol/l) with other major illness (e.g., seizures, critically unwell baby, severe respiratory distress, suspected infection with clinical instability or heart problem with cyanosis/poor perfusion or asphyxia with advanced resuscitation)	Buccal glucose gel Glucagon bolus i.m.	After 4 h if BG >36 mg/dl (>2.0 mmol/l) at both 1 and 4 h
Hypoglycemia of the newborn, Women’s Health Service, Christchurch Women’s (07/2020, New Zealand) (60)	- Preterm infants (GA <37 weeks) - SGA (<9^th^ centile (on UK-WHO growth chart) - LGA (>98^th^centile (on UK-WHO growth chart) - Hypothermia - Severe intrapartum fetal distress or lactate >5.8 mmol/L - Asymmetric growth in conjunction with either intrapartum fetal distress and/or meconium exposure - Unwell baby - Sepsis - Symptoms	- Maternal diabetes	pre-feed 3-4 h after birth	<48 h: <47 mg/dl (<2.6 mmol/l)	Yes	Not described in detail only when to contact the neonatal unit	Dextrose gel	One BG <47 mg/dl (<2.6 mmol/l): Until 3 consecutive BG are ≥47 mg/dl (≥2.6 mmol/l) without top-ups or dextrose gel. If a baby has always had BG of 47 mg/dl (2.6 mmol/l) or more and the feeding regime changes, i.e., from breastfeeds with top-ups to fully breastfeeding a pre-feed BG measurement is recommended 6-8 h after the last top-up
Swedish national guideline for prevention and treatment of neonatal hypoglycemia in newborn infants with gestational age ≥ 35 weeks (07/2019, Sweden) (55)	- Preterm infants (GA <37 weeks) - SGA (BW <-2 SDS) - LGA (BW >+2 SDS) - Sick infants at the NICU (e.g., asphyxia, infection) - Clinical signs of hypoglycemia	- Infants of diabetic mothers/mothers with GDM	Before the 2^nd^ feed (not later than 3 h after birth)	<72 h: <47 mg/dl (<2.6 mmol/l)	Yes, but only for severe symptoms (apnea, seizures etc.)	<27 mg/dl (<1.5 mmol/l) or <47 mg/dl (<2.6 mmol/l) and serious symptoms (apnea, seizures, impaired consciousness) or if hypoglycemia persists (27-34 mg/dl) (1.5-1.9 mmol/l) after one (to two) meals of supplementary feeds/dextrose gel	Buccal dextrose gel	Not described in detail
The screening and management of newborns at risk for low blood glucose (05/2019, Canada) (59)	- Preterm infants (GA <37 weeks) - SGA (BW <10^th^ centile) - LGA (BW >90^th^ centile) - Asphyxiated infants	- Infants of mothers with diabetes	At 2 h of age	<72 h: <47 mg/dl (<2.6 mmol/l)	Yes	<32 mg/dl (<1.8 mmol/l) or infants who have failed to respond to enteral supplementation	Dextrose gel	Preterm infants and SGA: after 24 h if BG ≥47 mg/dl (≥2.6 mmol/l) Maternal diabetes and LGA: after 12 h if BG ≥47 mg/dl (≥2.6 mmol/l)
Management of hypoglycemia in newborn: Turkish Neonatal and Pediatric Endocrinology and Diabetes Societies consensus report (2018, Turkey) (58)	- Prematurity - IUGR - SGA - LGA - Hypothermia - Perinatal asphyxia - Meconium aspiration syndrome - Infection - Polycythemia - Drug usage (IV indomethacin) - Immune hemolytic disease (Rh incompatibility) - Congenital heart diseases - Endocrine disorders - Special feature on physical examination findings - History of sibling with hypoglycemia - Malnutrition	- Maternal diabetes - Preeclampsia/eclampsia, gestation-related hypertension - Medical treatment (beta-blockers, oral hypoglycemic agents, beta-agonist tocolytics, late antepartum and intrapartum dextrose	30 min after first feed	<4 h: ≤40 mg/dl (≤2.2 mmol/l) 4-24 h: ≤45 mg/dl (≤2.5 mmol/l) >24 h: <50 mg/dl (<2.8 mmol/l)	Yes	Symptomatic and <40 mg/dl (<2.2 mmol/l) 0-4 h asymptomatic: BG <25 mg/dl (<1.4 mmol/l) twice despite feeding after first BG <25 mg/dl (<1.4 mmol/l) 4-24 h asymptomatic: BG <35 mg/dl (<1.9 mmol/l) twice despite feeding after first BG <35 mg/dl (<1.9 mmol/l)	Recommendations only if hyperinsulinism is diagnosed	Late preterm (34-36 6/7) and SGA: after 24 h Maternal diabetes and LGA (>34 weeks): after 12 h
Identification and Management of Neonatal Hypoglycemia in the Full Term Infant Framework for Practice, British Association of Perinatal Medicine (04/2017, UK) (54)	- IUGR (BW 2^nd^ centile) or clinically wasted - Perinatal acidosis (cord arterial or infant pH <7.1 and base deficit ≥-12mmol/l) - Hypothermia (<36.5°C) not attributed to environmental factors - Suspected/confirmed early onset sepsis - Abnormal feeding behavior - Clinical signs of hypoglycemia (cyanosis, apnea, altered level of consciousness, seizures, hypotonia, lethargy, high pitched cry)	- Infants of diabetic mothers - Infants of mothers taking beta-blockers in the third trimester and/or at time of delivery	Before the 2^nd^ feed (2-4 h after birth)	<48 h: <36 mg/dl (<2.0 mmol/l)	Yes	<18 mg/dl (<1.0 mmol/l) and/or clinical signs consistent with hypoglycemia	Buccal dextrose gel Glucagon i.m. (single administration)	BG ≥36 mg/dl (≥2.0 mmol/l) after 3^rd^ measurement (age <8 h). One BG 18-34 mg/dl (1.0-1.9 mmol/l): after 2 consecutive pre-feed BG measurements >2.0 mmol/l and no clinical signs. One BG <18 mg/dl (<1.0 mmol/l): continue to monitor BG until infant is on full enteral feeds and BG values are >45 mg/dl (>2.5 mmol/l) or 54 mg/dl (3.0 mmol/l) in cases of hyperinsulinism over several fast-feed cycles for at least 24 h
Clinical Report- Postnatal Glucose Homeostasis in Late-Preterm and Term Infants, American Academy of Pediatrics (2011, U.S) (5)	- Late preterm infants (GA 34-36 6/7) - SGA - LGA -	- Infants of mothers with diabetes	30 min after first feed	1-4 h: ≤40 mg/dl (≤2.2 mmol/l) 4-24 h: ≤45 mg/dl (≤2.5 mmol/l)	Yes	Symptomatic and BG <40 mg/dl (<2.2 mmol/l). Asymptomatic: Age <4 h: BG <25 mg/dl (<1.4 mmol/l) BG 25-40 mg/dl (1.4-2.2 mmol/l): refeed/i.v. glucose as needed. Age: 4-24 h: BG<35 mg/dl (<1.9 mmol/l). BG 35-45 mg/dl (1.9-2.5 mmol/l): refeed/i.v. glucose as needed.	–	Infants 34-36 6/7 weeks and SGA: after 24 h if BG ≥45 mg/dl (≥2.5 mmol/l). Maternal diabetes and LGA ≥34 weeks: 12 h if BG ≥45 mg/dl (≥2.5 mmol/l).
National guideline to prevent neonatal hypoglycemia (2010, Denmark) (63)	Low risk: - LGA (BW >+2SD*); >+22%) Moderate risk: - SGA (BW<-2SD); <-22%) - IUGR/Immaturity - Preterm (GA 32 + 0 - 36 + 6) - Sepsis, cooling - Light asphyxia (cord-pH 7.0-7.1 or BE -10 to -15) High risk: - Severe asphyxia (cord-pH <7.0 or BE <-15) - Severe IUGR/SGA (BW<-3SD); <-35%)	Low risk: - Diet-treated diabetes mother Moderate risk: - Insulin treated diabetic mother (sufficiently treated) High risk: - Diabetic fetopathies (insulin treated diabetic mother, dysregulated)	Low risk: 2 h Moderate risk: 2 h after first breastfeeding/ nutrition High risk: 1 h old	Depending on risk and age	No	<32 mg/dl (<1.8 mmol/l) Or repeatedly low, depending on the severity and the number of measurements	Diazoxide	Low risk: if first BG ≥2.5 mmol/l

BE, base excess; BWS, Beckwith-Wiedemann syndrome; GA, gestational age; BG, blood glucose; BW, birth weight; SCBU, special care baby unit; SGA, small for gestational age; LGA, large for gestational age; h, hour/s; i.v., intravenous; i.m., intramuscular; NICU, neonatal intensive care unit; SDS, Standard Deviation Score.

### Existing guidelines: preventive measures

While most guidelines place a clear focus on preventive measures to avoid hypoglycemia, there are also guidelines that do not address this (5). The majority however recommend initiating breastfeeding/feeding as early as possible (51, 52, 54–60, 62, 63). In addition, some emphasize that mothers should receive adequate guidance and support for breastfeeding and breastfeeding/feeding should be assessed regularly (51, 54, 55, 60, 62). Some guidelines recommend keeping intervals between feedings below three hours (51, 52, 54, 55, 57, 58, 60, 62) while others recommend breastfeeding ad libitum as long as no relevant hypoglycemia occurs (56, 59). Supplemental feeding with formular milk is recommended by Wackernagel et al. for a subgroup of infants in risk (e.g. preterm infants 35 + 0-3 + 6 weeks of gestation, small for gestational age (SGA), large for gestational age (LGA) with maternal diabetes, maternal diabetes not controlled by diet and sick infants in the NICU) and they recommend to use cup feeding or tube feeding instead of bottle feeding if possible (55). The Swiss Society of Neonatology Guideline also recommends offering formula milk immediately after breastfeeding until the mother has enough breast milk to sufficiently feed the neonate, and a prophylactic dose of buccal dextrose gel at one hour after birth (57). Oral dextrose gel (40%) might reduce the need for intravenous fluids in at-risk neonates and decrease NICU admissions with asymptomatic hypoglycemia (64–66), depending on the underlying guideline. In addition, an effect on successful implementation of breastfeeding was described (66). However, more data on safety and efficacy and the effect of dextrose gel on long-term neurological outcome are needed. In our view, there is no reliable data on further specific preventive measures. Of note, such measures must also be meaningfully embedded in the overall guideline. Common recommendations are to establish safe skin-to-skin contact immediately after birth, to keep the neonate warm and to facilitate breastfeeding (50, 52, 54–57, 60, 62). In our opinion, a guideline should also aim to reduce the burden of treatment to the least possible. Therefore, less burdensome measures such as the use of dextrose gel and the short-term use of formula milk might be considered helpful in reducing the need for more invasive procedures in at-risk neonates.

### Existing guidelines: who to screen?

Recommendations on which children should receive a blood glucose screening vary considerably between guidelines, ranging from four (5) to >25 risk factors (56). The only risk factor for which blood glucose screening is uniformly recommended is maternal diabetes (5, 50–57, 59, 60, 62, 63). Furthermore, there is a consensus that low birth weight (BW) infants are at increased risk for hypoglycemia, but there are different definitions for “low BW” or “SGA” depending on the guideline (e.g. BW <- 2 Standard Deviation Score (SDS) (55), BW ≤2^nd^ centile (54), BW <10^th^ centile (59), BW <2500 g or <3^rd^ centile (57)). The same applies to neonates born with a “high BW” or “LGA”. Definitions range from BW >90^th^ centile (59) or BW >+2 SDS (55) to BW >4.500 g (52) etc.

The difficulty of assessing the evidence for selective screening is exemplified on the basis of the study by Brand et al. who investigated the neurodevelopmental outcome at the age of 4 years in 75 healthy term LGA infants with transient hypoglycemia, that were born to non-diabetic mothers (67). The lowest blood glucose levels observed during the first five hours was 0.6 mmol/l (11 mg/dl) and the mean was 1.9 mmol/l (34 mg/dl). Of note, twenty-seven children (36%) were treated with intravenous glucose infusion and their mean blood glucose concentration was 1.4 mmol/l (25 mg/dl). With this intensive therapeutic approach, the neurological outcome for LGA-infants with hypoglycemia was not worse than without hypoglycemia. The conclusion of the authors is that transient mild hypoglycemia in LGA neonates does not to appear harmful – although this has been shown only in a cohort in which a significant proportion received i.v. glucose. Furthermore, exclusion of LGA newborns from screening would pose a risk to neonates with congenital hyperinsulinism, who are often LGA, and thus may be underestimated and identified too late, increasing the risk of severe brain damage for this patient population (32, 33).

For some potential risk factors evidence is low. For example, for neonates with polycythemia, which is listed as a risk factor in the Queensland guideline, the Turkish guideline and by Wight et al. (51, 56, 58). Hopfeld-Fogel et al. found that neonatal hypoglycemia was not more common compared to controls with normal hematocrit (68). On the other hand, the risk factor maternal beta-blocker treatment is only listed in some guidelines (50, 51, 54, 58, 62), although a large meta-analysis recently found that the risk of hypoglycemia in neonates of mothers treated with beta-blockers can be demonstrate with moderate certainty of evidence. Accordingly, the authors recommend a postnatal glycemic monitoring during the first 24 hours (69). Further assessment of the detailed evidence for the entire set of risk factors is not possible within the scope of this review. However, it is an important topic for separate meta-analyses or systematic reviews.

### Existing guidelines: blood glucose screening

No consensus exists on the optimal timing for the first postnatal blood glucose measurement. Most guidelines recommend blood glucose testing before the 2^nd^ feed but no later than 3 to 4 hours of age (51, 52, 54, 55, 57). Other recommendations range from 1 hour of age (50) to 30 minutes after the first feed (5, 58) or the time points differ depending on the risk factor (56, 63).

Hypoglycemia thresholds for neonates below 48/72 hours of age range from <2.0 mmol/l (<36 mg/dl) to <2.6 mmol/l (<47 mg/dl). Interestingly, 10 of the 13 reported guidelines (**Table 1**) differentiate between symptomatic and asymptomatic hypoglycemia in their recommendations for further management. However, as mentioned above it was found that the recognition of hypoglycemia signs is very observer dependent and “that it is difficult to distinguish the nonspecific signs of normal adaptation from signs of hypoglycemia” (4). Similarly, just as glucose thresholds vary widely, the further procedures in the event of hypoglycemia, e.g., when to start intravenous glucose, varies by guideline (**Table 1**). Some guidelines include further potential therapeutic measures such as buccal dextrose/glucose gel (50, 51, 54–57, 59, 60, 62), a glucagon bolus (50, 52, 54, 62) or continuously administered glucagon (51). In some guidelines, the duration of screening varies according to the risk factor (5, 56, 58, 59, 63), in others, it is based on the severity of hypoglycemia (54, 60). Both seem to make sense to us. An important aspect is also the recognition of severe hypoglycemic disorders and under which conditions these must be thought of or when one can safely discharge newborns with hypoglycemia without overlooking a persistent or transient hypoglycemic disease (7). We believe it is necessary for guidelines to include recommendations such as a reasonably long “safety” fasting test before discharge in neonates with hypoglycemia persisting beyond 48/72 hours, or in neonates with suspected or confirmed hypoglycemic disease (50–52, 56, 58, 59). The evidence of “how to screen” is very low, and therefore today a pragmatic approach must be chosen that also clarifies when glucose screening should be discontinued.

## Discussion, authors conclusions and implications for future research

Taken together, there is still insufficient data to define how low a glucose concentration must be, respectively, how long it must persist at which level to cause brain injury in neonates. Furthermore, the vulnerability for adverse consequences of hypoglycemia may vary for different context factors, such as immaturity, predisposing risk factors, availability of alternative fuels etc.

Comparing different guidelines worldwide, it becomes clear that the insufficient data basis leads to considerably different interpretation and management recommendations.

Thus, further studies on these questions are needed and have been demanded by experts for years. Ideas for ideal study designs have been proposed including studies in prospective cohorts with nested randomized treatment trial or randomized trials of different approaches to prevention or screening and diagnosis of hypoglycemia in at-risk neonates (38, 70, 71).

Until data from such studies is available, the main question is how to interpret available evidence and follow the principle of “primum non nocere” in terms of prevention, screening strategy and treatment.

### Should selective screening of blood glucose in at-risk newborns be adhered to and who is at risk?

Neonatal hypoglycemia screening affects a high number of neonates - depending on the definition, approximately one third of newborns have at least one risk factor for hypoglycemia. The diagnosis of hypoglycemia has relevant implications for management and care of neonates. In one recent study, 529 of 10,533 infants were admitted to the NICU postpartum, and of those, almost half (n= 235, 44,4%) for hypoglycemia management (72).

However, it has been postulated recently that screening for neonatal hypoglycemia does not meet the principles for a screening test (73). It was discussed that the screening may cause harm and that the current screening approach does not prevent severe hypoglycemia and severe brain damage.

Despite the paucity of evidence for any specific evidence-based approach, it is without doubt that there is a considerable number of children suffering from hypoglycemic brain injury because of insufficient screening and management strategies – e.g., those with inborn hypoglycemia disorders such as congenital hyperinsulinism. Furthermore, it is also without doubt that also transient hyperinsulinemic hypoglycemia without predisposing risk factors may cause hypoglycemic brain injury of varying severity (32, 34). The authors advocate that – *particularly* as there is limited evidence on timing and interventional cut-offs for screening and treatment - we owe the affected newborns balanced and thoughtful guidelines to the best of our knowledge, to be diagnosed timely and treated adequately to prevent adverse outcomes that negatively affect their lives permanently.

Preventive and screening strategies, as well as therapeutic efforts, impose only a temporary and usually minor burden. Therefore, the authors advocate accepting some degree of overtreatment to prevent long-term impairment in some - similar to the screening and treatment of neonatal hyperbilirubinemia, which implies a significant number of interventions on patients that might not suffer from negative consequences even untreated, to reliably identify and treat those who are at immediate risk for hyperbilirubinemic brain damage (74).

In newborns at risk for hypoglycemia, blood glucose should be checked according to a predefined schedule, usually starting from 2-3 hours of life. As summarized before, published recommendations vary considerably regarding the risk factors that should trigger systematic screening, and regarding the interventions. At least for the most important risk factors SGA/fetal growth restriction (FGR), maternal diabetes, preterm birth, and other forms of perinatal stress, clear recommendations for screening are needed, based on currently available data. However, predicting postnatal blood glucose concentrations is not straightforward in infants at risk (47) and it is unclear which neonates with other risk factors may need to be screened for neonatal hypoglycemia. Certainly, it seems to be justified for other risk factors, such as maternal beta-blocker therapy.

### Which therapeutic measures are appropriate in which situations?

Management of at-risk newborns does not start with blood glucose screening. Preventive measures can be certainly effective without causing harm, including frequent feeding, keeping the newborn warm, and ensuring safe skin-to-skin contact – these measures should be made available to all at-risk newborns. Moderate measures to prevent or treat hypoglycemia can be supplemental formula feeding or dextrose gel application – both with very limited negative consequences. However, invasive measures such as intravenous glucose, glucagon treatment, or transfer to a NICU should be recommended thoughtfully and restrictively in a guideline, but then put into practice consistently with stepwise rapid escalation of treatment when indicated to reliably prevent severe and persistent hypoglycemia with potentially adverse consequences.

Interventions are e.g. recommended when the blood glucose concentration falls below a critical threshold and does not rise above this value, or when symptoms are observed (46). Commonly, below 2.5 mmol/l (45 mg/dl) “action” is recommended in any newborn with signs attributed to hypoglycemia (46). However, based on blood glucose levels alone, there is some debate if action should be initiated at threshold values <2.0 mmol/l or <2.5 mmol/l (<36 mg/dl or <45 mg/dl). While a single blood glucose measurement between 2.0 and 2.5 mmol/l (36 and 45 mg/dl) might not make a big difference, low values that occur repeatedly (indicating a certain severity), are accompanied by symptoms (indicating neuroglycopenia), or occur during a period of insufficient feeding (indicating limited potential for spontaneous recovery) are of greater concern. In terms of symptoms, it should be considered that insufficient feeding despite low glucose values could indicate apathy caused by neuroglycopenia.

We recommend the operational threshold and the target blood glucose value to aim for should be >2.5 mmol/l (>45 mg/dl) from at least the 4^th^ (-6^th^) hour of life (75). This higher threshold seems more appropriate to us to provide a margin of safety and to safely avoid blood glucose levels <1.7 mmol/l (<30 mg/dl). Especially prolonged periods or repeated periods in this range must be considered potentially harmful and a value that triggers invasive measures.

### How are neonates with relevant hypoglycemia identified among neonates without known risk factors?

Because routine blood glucose screening is usually not performed in asymptomatic, healthy, term newborns, neonates with transitory or transient hypoglycemia as well as a permanent hypoglycemic disease, who do not have any risk factors for hypoglycemia are often first noticed by clinical signs (32). Therefore, without a general glucose screening, at least careful education and awareness of parents, midwives, and nurses regarding the clinical signs of hypoglycemia is needed. When a neonate presents with signs suggestive of hypoglycemia, e.g., adrenergic or neuroglycopenic symptoms, it is consensus that a blood glucose determination should be performed. However, given the limited sensitivity and specificity of symptoms, in case of doubt regarding signs of low glucose, only a blood glucose measurement can reliably detect or exclude hypoglycemia, and should therefore be performed quite generously (“glucose as a vital sign”).

Regarding missing the early diagnosis of congenital hypoglycemic disorders or transient hyperinsulinism in the neonatal period, it is important that treatment standards define criteria for when to consider such a condition e.g., severity and/or duration of hypoglycemia, carbohydrate requirement etc. Today, these newborns often receive appropriate treatment for severe hyperinsulinism, but only after a significant delay (32). However, criteria should also be defined for the termination of preventive, screening, or therapeutic measures when there is no longer a relevant risk for recurrent or severe hypoglycemia. In unclear cases, a short diagnostic fasting test over 5-6 hours may also be helpful to exclude further hypoglycemic risk.

### Future research

Severe hypoglycemic brain damage may not occur in many patients classified as having transitory hypoglycemia in the first days of life. More likely is the occurrence of minor intelligence reduction or partial performance deficits which may manifest too late in childhood to be consciously attributed to neonatal hypoglycemia (38). It can be assumed that at-risk newborns are particularly susceptible because they have, depending on the condition, low energy reserves overall, but especially in the brain. In addition, it seems likely that their limited adaptive capacities can only provide insufficient energy in the form of glucose, lactate, or ketone bodies. To prove this in studies, clinically and metabolically well characterized at-risk newborns, must be followed up neurologically in detail and on a long-term basis. Lower thresholds beyond 2.5 mmol/l (45 mg/dl) should be investigated as primary outcome to see if these are associated with worse outcomes. In particular, the duration and the frequency of hypoglycemia should be included in the analysis.

In parallel, guidelines should be prospectively evaluated in terms of efficacy regarding glycemic control and long-term neurological outcome at least until mid-childhood, when partial performance disorders become apparent. The studies should be powered to detect even small reductions in neurodevelopmental outcomes. On a population level, even small effects, e.g., a quarter of a standard deviation rather than half a standard deviation, as frequently used in studies, can be highly clinically relevant. Unnecessary overtreatment should also be avoided, and a long-term goal should be to establish evidenced-based guidelines that provide an appropriate approach for the entire neonatal population. These demands result in very challenging study designs in terms of the number of subjects and study duration. On the other hand, the topic is of such high relevance that this high effort seems justified. Authors’ recommendation for future studies are summarized in **Table 2**.

**Table 2:** Recommendations on possible targets for future studies to increase evidence for the management of neonatal hypoglycemia.

• Prospective, preferably multicenter randomized controlled trials evaluating long-term outcome until at least mid-childhood with power to detect even small reductions in neurodevelopmental outcomes depending on: o different risk factors (between risk factors and within a specific risk factor group) o different operational thresholds (overtreatment vs. undertreatment) o duration and frequency of hypoglycemia o different preventive and treatment methods (e.g. guidelines) o early identification of inborn endocrine or metabolic disease causing severe neonatal hypoglycemia o symptomatic/asymptomatic hypoglycemia o the influence of alternative cerebral energy fuels (e.g. ketones, lactate) • Prospective cohort studies evaluating the individual risk for severe hypoglycemia according to different risk factors and development of risk factor specific screening and treatment approaches • Randomized controlled trials of signs of hypoglycemia: Does training of parents, midwives, and nurses affect early detection of severe hypoglycemia?

## Author contributions

All authors listed have made a substantial, direct, and intellectual contribution to the work and approved it for publication.
